# 抗CD19 CAR-T疗法治疗弥漫大B细胞淋巴瘤失败患者的挽救性治疗的临床疗效及安全性分析

**DOI:** 10.3760/cma.j.cn121090-20260413-00193

**Published:** 2026-07

**Authors:** 燕 陆, 莉莉 周, 世光 叶, 存影 姚, 波涛 王, 爱斌 梁, 萍 李

**Affiliations:** 1 同济大学附属同济医院血液科，上海 200065 Department of Hematology, Tongji Hospital, Tongji University School of Medicine, Shanghai 200065, China; 2 安徽医科大学第二附属医院血液科，合肥 230601 Department of Hematology, the Second Affiliated Hospital of Anhui Medical University, Hefei 230601, China; 3 河南省南阳医学高等专科学校第一附属医院，南阳 473000 Department of Hematology, The First Affiliated Hospital of Nanyang Medical College, Nanyang 473000, China

**Keywords:** 嵌合抗原受体T细胞, 靶向CD20 T细胞免疫治疗, 挽救性治疗, 复发/难治弥漫大B细胞淋巴瘤, Chimeric antigen receptor T cells, Anti-CD20 T-cell immunotherapy, Salvage therapy, Relapsed/refractory diffuse large B-cell lymphoma

## Abstract

**目的:**

探讨经抗CD19的嵌合抗原受体（CAR）-T疗法治疗失败的复发/难治弥漫大B细胞淋巴瘤（DLBCL）患者挽救性治疗的有效性和安全性。

**方法:**

本研究为单中心、回顾性、比较性队列研究。回顾性分析2022年3月至2026年3月在同济大学附属同济医院血液科接受抗CD19 CAR-T疗法治疗失败的23例DLBCL患者的临床资料。根据挽救治疗方案分为靶向CD20 T细胞免疫治疗组（12例）和非靶向CD20 T细胞免疫治疗组（11例），比较两组患者的疗效和安全性。

**结果:**

研究共纳入23例患者，中位随访13.5（95％*CI*：10.4～31.9）个月，客观缓解率（ORR）为60.8％（14/23），11例（47.8％）患者获完全缓解（CR）；中位无进展生存（PFS）时间未达到，中位总生存（OS）时间未达到。其中靶向CD20 T细胞免疫治疗组的ORR为100％，11例（91.7％）患者获得CR，PFS时间未达到，中位OS时间未达到；而非靶向CD20 T细胞免疫治疗组的ORR为18.2％（95％*CI*：2.3％～51.8％），仅2例患者获部分缓解（PR），中位PFS时间仅为1.7个月，中位OS时间仅为2.6个月；两组患者的PFS及OS差异均有统计学意义（均*P*<0.001）。在安全性方面，靶向CD20 T细胞免疫治疗组中有10例（83.3％）患者仅发生了1～2级细胞因子释放综合征，无免疫效应细胞相关中枢神经毒性综合征发生。非靶向CD20 T细胞免疫治疗组主要不良反应为血液学毒性。

**结论:**

在抗CD19 CAR-T疗法治疗失败的DLBCL患者的挽救性治疗中，靶向CD20 T细胞免疫疗法相较于非靶向CD20 T细胞免疫疗法显示出更高的缓解率和更长的生存期，且安全性可控。

## Abstract (en)

**Objective:**

To evaluate the efficacy and safety of the salvage treatment in patients with relapsed/refractory diffuse large B-cell lymphoma（DLBCL）who failed prior anti-CD19 chimeric antigen receptor T-cell（CAR-T）therapy.

**Methods:**

This study retrospectively analyzed clinical data of 23 patients with DLBCL who failed CD19 CAR-T therapy at the Department of Hematology, Tongji Hospital, from March 2022 to March 2026. Patients were divided into the anti-CD20 T-cell immunotherapy group（*n*＝12）and non-anti-CD20 T-cell immunotherapy group（*n*＝11）. Efficacy and safety were compared between the two groups.

**Results:**

This study included 23 patients. With a median follow-up of 13.5（95％*CI*: 10.4–31.9）months, the overall response rate（ORR）was 60.8％（14/23）, and 11（47.8％）patients achieved complete remission（CR）. Median progression-free survival（PFS）and overall survival（OS）were not reached. In the anti-CD20 T-cell immunotherapy group, ORR was 100％ with a CR rate of 91.7％, and the median PFS and OS were not reached. In the non-anti-CD20 T-cell immunotherapy group, ORR was 18.2％（95％ *CI*: 2.3％–51.8％）with only two partial responses, and the median PFS and OS were 1.7 and 2.6 months, respectively（*P*<0.001 for both）. Regarding safety, 10（83.3％）patients in the T-cell immunotherapy group developed grade 1–2 cytokine release syndrome, and no immune effector cell-associated neurotoxicity syndrome occurred. In the non-anti-CD20 T-cell immunotherapy group, the main adverse events were hematologic toxicities.

**Conclusion:**

Anti-CD20 T-cell immunotherapy yields significantly higher response rates and longer survival compared with non-anti-CD20 T-cell immunotherapy in patients with DLBCL failing anti-CD19 CAR-T therapy, with a manageable safety profile.

弥漫大B细胞淋巴瘤（diffuse large B cell lymphoma, DLBCL）是最常见的成人淋巴造血系统恶性肿瘤，具有侵袭性强、异质性高的特点。尽管50％～70％的患者可通过标准一线免疫化疗获得长期缓解，但仍有约40％的患者原发难治或缓解后复发，预后极差，复发后中位总生存（overall survival, OS）时间仅6个月，2年OS率约20％[1]–[2]。自2017年以来，靶向CD19的嵌合抗原受体T细胞（chimeric antigen receptor T cell, CAR-T）疗法获批用于治疗复发/难治性（relapsed/refractory, r/r）DLBCL，成为该领域的重要突破。虽然CAR-T疗法在r/r DLBCL患者中可获得53％～80％的客观缓解率（objective response rate, ORR），但约60％的患者在输注后2年内出现疾病进展，且进展后OS时间明显缩短[2]–[5]。针对CAR-T治疗失败后患者的挽救性治疗（包括化疗、抗体药物偶联物、分子靶向药物、放疗、异基因造血干细胞移植等）的ORR和完全缓解（complete remission, CR）率分别仅为8％～54.1％和3.8％～35％，中位无进展生存（progression-free survival, PFS）和OS时间分别为1.4～3.8个月和3.8～9.3个月[6]–[12]。

CD20在超过95％的正常B细胞和B细胞来源的肿瘤细胞表面特异性表达，是B细胞恶性肿瘤免疫治疗的理想靶点[13]。近年来，靶向CD20的T细胞免疫疗法（anti-CD20 T-cell immunotherapy）包括CAR-T细胞治疗和双特异性抗体治疗在B细胞淋巴瘤中显示出良好疗效[14]–[17]。一项抗CD20 CAR-T（C-CAR066）在抗CD19 CAR-T治疗失败的r/r 大B细胞淋巴瘤（large B cell lymphoma, LBCL）患者的Ⅰ期研究结果显示，ORR高达92.9％，CR率为57.1％，安全性良好[18]。靶向CD20×CD3的双特异性T细胞衔接抗体格菲妥单抗，在LYSAⅡ期临床试验（NCT04703686）中，用于CAR-T治疗失败后的r/r DLBCL患者，中位随访15.3个月，最佳总缓解率（overall metabolic response rate, OMRR）为76.1％，最佳完全代谢缓解率（CMRR）为45.7％。中位OS时间达14.7（90％*CI*: 8.8～未达到）个月，中位PFS时间为3.8（95％*CI*: 2.4～19.6）个月[19]。而抗体偶联药物（如维泊妥珠单抗、泰朗妥昔单抗）在CAR-T失败后的ORR为8％～54.1％，中位PFS时间仅1.4～3.8个月，疗效有限[20]–[22]。

本研究回顾性分析同济大学附属同济医院血液科收治的23例抗CD19 CAR-T疗法失败的r/r DLBCL患者，比较靶向CD20 T细胞免疫治疗组（抗CD20 CAR-T/双特异性抗体）与非靶向CD20 T细胞免疫治疗组（抗体偶联药物、分子靶向药物联合传统化疗）的临床疗效和安全性，现报道如下。

## 病例与方法

一、病例

本研究为一项单中心、回顾性、比较性队列研究。纳入23例2022年3月至2026年3月在同济大学附属同济医院血液科经CD19 CAR-T治疗失败的伴有高危特征的r/r DLBCL患者。所有入组患者均伴有至少一项以下高危特征：结外受累、Ann Arbor Ⅲ/Ⅳ期、乳酸脱氢酶升高（>250 U/L）或美国东部肿瘤协作组体能状况评分（Eastern Cooperative Oncology Group, ECOG评分）≥3分。

入组标准：①年龄≥18岁，经组织学及免疫组化确诊为CD20阳性DLBCL；②既往曾接受过已上市的抗CD19 CAR-T治疗，并具有足够的器官和骨髓功能；③至少有一个可评估病灶。排除标准：①合并活动性自身免疫病；②合并严重感染或感染急性期。本研究获得同济大学附属同济医院伦理委员会批准［伦理批号：（同）伦审第（2022-065）号］。所有患者均签署了知情同意书。

二、治疗方法

23例患者均进行了CD19 CAR-T后的挽救性治疗。本研究为回顾性研究，由临床医师根据患者的临床一般情况，评估细胞免疫治疗的可行性，可能存在一定的选择偏倚。12例患者应用了靶向CD20 T细胞免疫疗法（10例抗CD20 CAR-T，2例抗CD3×CD20的双特异性抗体）；11例患者为非靶向CD20 T细胞免疫治疗组，接受了抗体偶联药物、分子靶向药物联合多药化疗的治疗。

10例进行抗CD20 CAR-T治疗的患者均接受了氟达拉滨（25 mg·m^−2^·d^−1^，−5～−3 d）和环磷酰胺（300 mg·m^−2^·d^−1^，−5～−3 d）清除淋巴细胞预处理。第0天开始，通过静脉输注总剂量为3×10^6^/kg的抗CD20 CAR-T细胞（共刺激分子均为4-1BB），1次输注完成。

应用抗CD3×CD20的双特异性抗体疗法患者中，1例为格菲妥单抗联合维泊妥珠单抗，第1周期给予奥妥珠单抗1 000 mg（第1天）、维泊妥珠单抗1.8 mg/kg（第2天）及格菲妥单抗递增剂量给药（2.5 mg，第8天；10 mg，第15天）；第2周期起给予格菲妥单抗30 mg（第1天）联合维泊妥珠单抗1.8 mg/kg（第1天），每3周为1个治疗周期。维泊妥珠单抗共给予6个周期，格菲妥单抗共给予12个周期。另1例（中枢复发患者）接受格菲妥单抗联合替莫唑胺及匹妥布替尼治疗。第1周期给予奥妥珠单抗1 000 mg（第0天）、替莫唑胺100 mg·m^−2^·d^−1^（第1～5天）、匹妥布替尼200 mg/d，以及格菲妥单抗递增剂量给药（2.5 mg，第7天；10 mg，第14天）；第2周期起给予格菲妥单抗30 mg（第1天）联合替莫唑胺100 mg·m^−2^·d^−1^（第1～5天）及匹妥布替尼200 mg/d，每3周为1个治疗周期，共治疗5个周期。

11例非靶向CD20 T细胞免疫治疗组患者，具体治疗方案为分子靶向药物±利妥昔单抗±维泊妥珠单抗联合多药化疗。塞利尼索40 mg每周1次；西达本胺20 mg每周2次；利妥昔单抗375 mg/m^2^，d 0；维泊妥珠单抗1.8 mg/kg，第1天。多药化疗方案主要包括：（1）ICE（2例）：依托泊苷（100 mg·m^−2^·d^−1^，第1～3天）+卡铂（最大剂量800 mg，第2天）+异环磷酰胺（5 g/m^2^，第2天）；（2）ESHAP（1例）：依托泊苷（60 mg·m^−2^·d^−1^，第1～4天）+甲泼尼龙（500 mg·m^−2^·d^−1^，第1～3天）+阿糖胞苷（2 g/m^2^，每12 h 1次，第5天）+顺铂（25 mg·m^−2^·d^−1^，第1～4天）；（3）GDP（2例）：吉西他滨（1 g·m^−2^·d^−1^，第1、8天）+地塞米松（40 mg/d，第1～4天）+顺铂（75 mg/m^2^，第1天）；（4）GMOEX（6例）：吉西他滨（1 g·m^−2^·d^−1^，第1天）+奥沙利铂（100 mg/m^2^，第1天）。每21 d为1个治疗周期。

三、随访与疗效评价

挽救性治疗后1、2、3、6、9、12个月行增强CT检查，3、6、12个月行PET-CT检查评估治疗效果。淋巴瘤疗效评价标准参照Lugano淋巴瘤疗效评估标准[23]。通过评估可测量病灶最大垂直径乘积之和（sum of products of greatest diameters, SPD）缩小情况反应经挽救性治疗后肿瘤负荷变化情况。细胞因子释放综合征（cytokine release syndrome, CRS）及免疫效应细胞相关中枢神经毒性综合征（immune effector cell-associated neurotoxicity syndrome, ICANS）的定义和分级根据《CD19 CAR-T治疗B-NHL毒副作用临床管理中国专家共识（2022版）》[24]进行判定。其他不良反应采用不良事件通用术语标准（common terminology criteria for adverse events, CTCAE）5.0版分级标准进行判定。随访截止时间为2026年3月，中位随访13.5（95％*CI*：10.4～31.9）个月。全组最短随访时间为1.6个月（2026年1月入组），最长为39个月。仅最后入组这1例患者随访时间不足3个月，且该患者仍在持续随访中。PFS时间定义为自患者开始行挽救性治疗至疾病进展、复发、死亡或随访截止时间，OS时间定义为自患者开始行挽救性治疗至死亡或末次随访的时间。

四、统计学处理

采用GraphPad Prism 9.0进行数据描述及可视化。连续变量以中位数（范围）进行描述，分类变量以例数（构成比）进行描述；所有患者均纳入生存分析，通过Kaplan-Meier法进行生存分析，中位生存时间及1年生存率采用点估算值（95％ *CI*）进行描述。

## 结果

一、基线临床特征

共入组23例经CD19 CAR-T治疗失败伴有高危风险的r/r DLBCL患者，既往CD19 CAR-T最佳疗效CR 12例、部分缓解（PR）9例、疾病稳定（SD）2例。其中既往接受CD19 CAR-T治疗后CR患者的中位缓解持续时间（DOR）为6.4（95％*CI*：4.7～11.1）个月，总体患者的中位DOR为4.5（95％*CI*：3.0～6.4）个月。23例患者CD19 CAR-T治疗失败后的病理标本（淋巴结或组织活检）中，21例有可评估的CD19表达结果：18例CD19表达阳性，3例出现CD19完全丢失（靶向CD20 T细胞免疫治疗组2例，非靶向CD20 T细胞免疫治疗组1例）；所有患者CD20均为阳性。入组患者的中位年龄为59（33～78）岁，12例（52.2％）美国东部肿瘤协作组体能状况评分（ECOG评分）≥2分；12例（52.2％）为双表达DLBCL，靶向CD20 T细胞免疫治疗组有7例（58.3％）；5例（21.7％）为双打击DLBCL，靶向CD20 T细胞免疫治疗组有2例（16.7％）；18例（78.3％）国际预后指数（IPI）评分≥3分，靶向CD20 T细胞免疫治疗组占9例（75.0％）；18例（78.3％）患者存在结外受累，10例（83.3％）为靶向CD20 T细胞免疫治疗组患者；3例（13.0％）伴中枢神经系统累及，靶向CD20 T细胞免疫治疗组有2例（16.7％）中枢神经系统累及患者；11例（47.8％）伴有TP53缺失或突变，靶向CD20 T细胞免疫治疗组有8例（66.7％）。具体临床特征见表1。

**表1:** 23例经CD19 CAR-T治疗失败的复发/难治弥漫大B细胞淋巴瘤（DLBCL）患者的临床特征

临床特征	总体 （23例）	靶向CD20 T细胞免疫治疗组 （12例）	非靶向CD20 T细胞免疫治疗组 （11例）
年龄［岁，*M*（范围）］	59（33～78）	59（33～70）	64（43～78）
年龄≥65岁［例（％）］	8（34.8）	3（25.0）	5（45.5）
男性［例（％）］	15（65.2）	7（58.3）	8（72.7）
基础疾病［例（％）］			
糖尿病	4（17.4）	2（16.7）	2（18.2）
心脏病	6（26.1）	1（8.3）	5（45.5）
慢性阻塞性肺疾病	2（8.7）	2（16.7）	0（0）
IPI评分≥3分［例（％）］	18（78.3）	9（75.0）	9（81.8）
中位ECOG评分［*M*（范围）］	2（0～4）	2（0～4）	1（0～3）
Ki-67≥80％［例（％）］	15（65.2）	8（66.7）	7（63.6）
TP53缺失或突变［例（％）］	11（47.8）	8（66.7）	3（27.3）
原发难治［例（％）］	18（78.3）	10（83.3）	8（72.7）
既往治疗线数［*M*（范围）］	3（2～10）	3（2～10）	4（2～7）
治疗线数≥3线［例（％）］	16（69.6）	7（58.3）	9（81.8）
肿块直径≥5 cm［例（％）］	6（26.1）	2（16.7）	4（36.4）
DLBCL亚型［例（％）］			
双表达	12（52.2）	7（58.3）	5（45.5）
双打击	5（21.7）	2（16.7）	3（27.3）
结外受累［例（％）］	18（78.3）	10（83.3）	8（72.7）
伴中枢神经系统累及	3（13.0）	2（16.7）	1（9.1）
既往治疗方案［例（％）］			
自体造血干细胞移植	2（8.7）	1（8.3）	1（9.1）
放疗	8（34.8）	6（50.0）	2（18.2）
来那度胺	13（56.5）	5（41.7）	8（72.7）
西达本胺	7（30.4）	4（33.3）	3（27.3）
BTK抑制剂	15（65.2）	8（66.7）	7（63.6）
既往CD19 CAR-T最佳疗效［例（％）］			
完全缓解	12（52.2）	8（66.7）	4（36.4）
部分缓解	9（39.1）	3（25.0）	6（54.5）
疾病稳定	2（8.7）	1（8.3）	1（9.1）
CD19 CAR-T细胞共刺激因子［例（％）］			
CD28	14（60.9）	9（75.0）	5（45.5）
4-1BB	9（39.1）	3（25.0）	6（54.5）
挽救治疗前CD19表达［例（％）］	18（85.7）	9（81.8）	9（90.0）

**注** CAR-T：嵌合抗原受体T细胞；IPI评分：国际预后指数评分；ECOG评分：美国东部肿瘤协作组体能状况评分

二、疗效分析

11例（47.8％）患者获CR，均为靶向CD20 T细胞免疫治疗组；中位PFS期未达到，中位OS期未达到，1年PFS率为52.2％（95％*CI*：42.1％～84.1％），1年OS率为52.2％（95％*CI*：41.5％～83.9％）。靶向CD20 T细胞免疫治疗组患者ORR为100％，11例（91.7％）患者获CR，且所有输注CD20 CAR-T细胞患者都获得了CR，其中有4例患者靶病灶消失，中位PFS时间未达到，中位OS时间未达到；而非靶向CD20 T细胞免疫治疗组的ORR为18.2％（95％*CI*: 2.3％～51.8％），仅2例患者获PR，中位PFS时间仅为1.7个月，中位OS时间仅为2.6个月；两组患者的PFS及OS差异均有统计学意义（均*P*<0.001）（图1）。

**图1:**
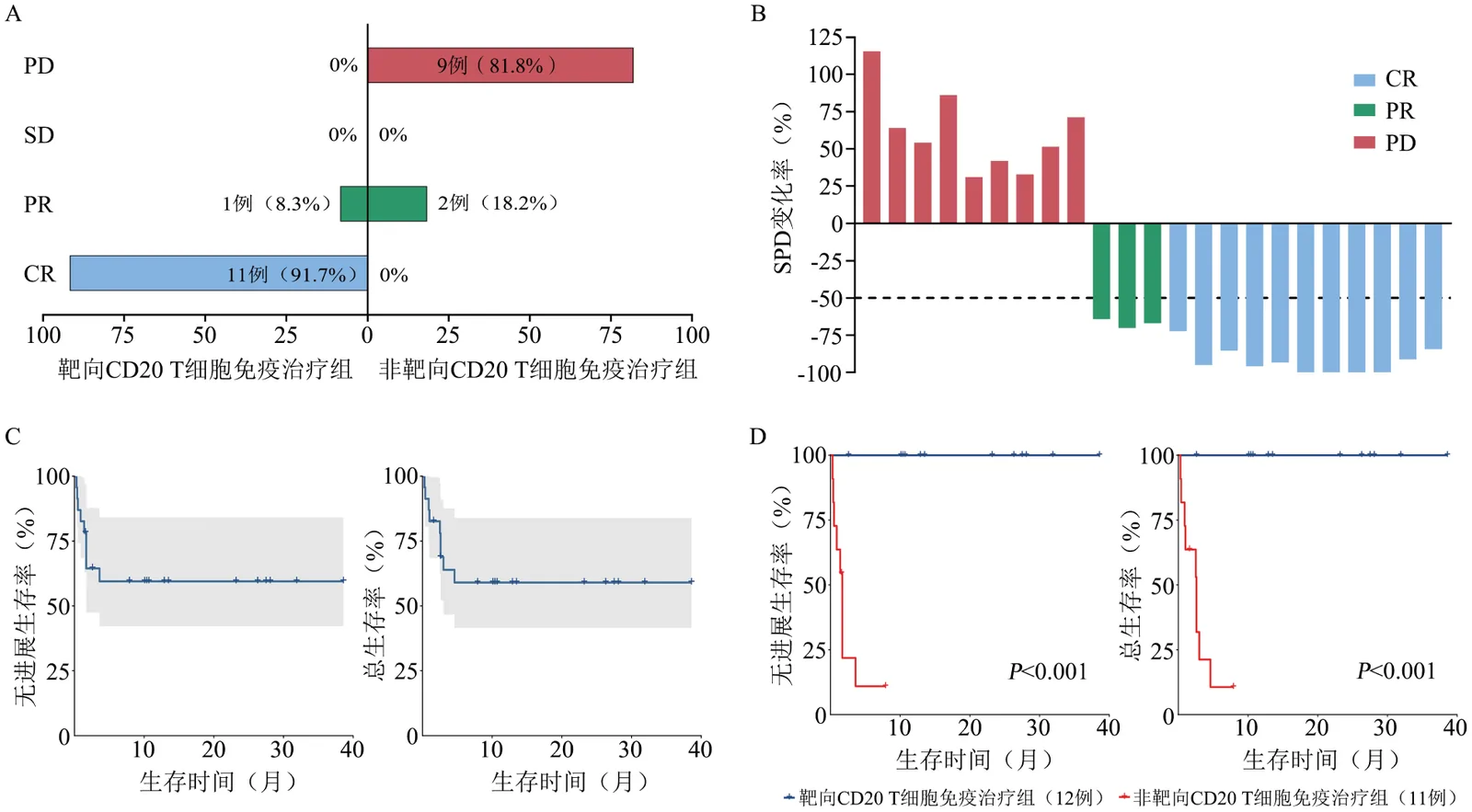
23例患者的挽救性治疗疗效分析 **A** 总反应率及两组对比；**B** 靶病灶体积（SPD）瀑布图；**C** 23例患者无进展生存时间（PFS）与总生存时间（OS）曲线；**D** 靶向CD20 T细胞免疫治疗组与非靶向CD20 T细胞免疫治疗组的生存时间（PFS）与总生存时间（OS）曲线 **注** PD：疾病进展；SD：疾病稳定；PR：部分缓解；CR：完全缓解

三、安全性

靶向CD20 T细胞免疫治疗组中10例（83.3％）患者发生了CRS，均为1～2级CRS，未发生≥3级CRS。9例（75.0％）出现1级CRS，1例（8.3％）出现2级CRS，无ICANS发生。所有12例患者均发生血液学不良反应，其中4级中性粒细胞减少9例（75.0％），4级血小板减少5例（41.7％），贫血为1～3级，3级贫血5例（41.7％）；4例（33.3％）患者出现轻度肝功能不全，仅2例（16.7％）患者轻度肌酐升高。12例患者中有5例（41.7％）出现凝血功能异常，均为1～2级（表2）。

**表2:** 靶向CD20 T细胞免疫治疗组的不良事件［例（％）］

不良事件	总体	1级	2级	3级	4级
细胞因子释放综合征	10（83.3）	9（75.0）	1（8.3）	0（0）	0（0）
ICANS	0（0）	0（0）	0（0）	0（0）	0（0）
发热	10（83.3）	9（75.0）	0（0）	1（8.3）	0（0）
低血压	1（8.3）	1（8.3）	0（0）	0（0）	0（0）
血氧饱和度下降	0（0）	0（0）	0（0）	0（0）	0（0）
贫血	12（100）	3（25.0）	4（33.3）	5（41.7）	0（0）
中性粒细胞减少	12（100）	0	1（8.3）	2（16.7）	9（75.0）
血小板减少	12（100）	2（16.7）	1（8.3）	4（33.3）	5（41.7）
肝功能损害	4（33.3）	3（25.0）	1（8.3）	0（0）	0（0）
凝血功能异常	5（41.7）	4（33.3）	1（8.3）	0（0）	0（0）
肾功能损害	2（16.7）	2（16.7）	0（0）	0（0）	0（0）

**注** ICANS：免疫效应细胞相关中枢神经毒性综合征

非靶向CD20 T细胞免疫治疗组主要不良反应为血液学毒性，以贫血、血小板减少、中性粒细胞减少、凝血功能异常为主。所有11例（100％）患者均出现不同程度的贫血，为1～3级，3级贫血8例（72.7％）；分别有6例（54.5％）与4例（36.4％）患者发生了4级血小板减少及中性粒细胞减少；10例（90.9％）患者出现了凝血功能异常，均为1～2级。2例（18.2％）患者出现3级肝功能不全；7例（63.6％）出现了1～2级的感染（表3）。

**表3:** 非靶向CD20 T细胞免疫治疗组的不良事件［例（％）］

不良事件	总体	1级	2级	3级	4级
中性粒细胞减少	8（72.7）	1（9.1）	0（0）	3（27.3）	4（36.4）
血小板减少	10（90.9）	0（0）	2（18.2）	2（18.2）	6（54.5）
贫血	11（100）	1（9.1）	2（18.2）	8（72.7）	0（0）
肝功能不全	8（72.7）	2（18.2）	4（36.4）	2（18.2）	0（0）
肾功能不全	2（18.2）	1（9.1）	1（9.1）	0（0）	0（0）
凝血功能异常	10（90.9）	5（45.5）	5（45.5）	0（0）	0（0）
感染	7（63.6）	1（9.1）	6（54.5）	0（0）	0（0）

## 讨论

本研究结果显示，在抗CD19 CAR-T治疗失败的r/r DLBCL患者中，采用靶向CD20 T细胞免疫疗法进行挽救治疗可获得极高的缓解率（ORR 100％，CR率91.7％），且缓解持久，中位PFS和OS时间均未达到；而非靶向CD20 T细胞免疫疗法治疗（抗体偶联药物、分子靶向药物联合多药化疗）疗效较差，ORR仅18.2％，中位PFS和OS时间分别仅1.7个月和2.6个月。这一结果与既往报道的CAR-T失败后挽救治疗疗效不佳的结论一致[9],[11],[21],[25]。例如，DESCAR-T注册研究显示，CAR-T治疗后进展的LBCL患者接受后续治疗的中位PFS和OS时间分别为2.8个月和5.2个月[9]。抗体药物偶联物如泰朗妥昔单抗（loncastuximab）和维泊妥珠单抗（polatuzumab vedotin）在CAR-T失败后的中位PFS时间分别为1.4个月和10周[11],[21]，帕博利珠单抗的ORR为25％[25]。这些数据进一步凸显了CAR-T失败后患者的治疗困境。

CD19 CAR-T治疗失败的机制复杂多样，CAR-T细胞的细胞动力学和肿瘤负荷是影响CAR-T细胞治疗的两个重要因素。CAR-T细胞的持久性是影响恶性血液病预后的重要因素。有患者在CD19 CAR-T治疗后数年内CD19 CAR转基因序列在其外周血内仍可检测到，这表明CAR-T细胞能够在体内存活很长一段时间[26]。此外，肿瘤负荷是影响CAR-T治疗疗效的另一个重要因素。推测AUC0-28d（从开始到注射后28 d的曲线下面积）与肿瘤负荷的比值可能可以预测患者的长期预后[27]。除了CAR-T细胞的细胞动力学和肿瘤负荷外，肿瘤细胞的存活取决于肿瘤微环境（TME）的状况，包括免疫细胞、细胞外基质、血管和基质细胞等。肿瘤微环境不仅仅是一个免疫抑制环境，也是肿瘤发生发展的积极促进者。一些共刺激和共抑制分子存在于TME中，并相互作用以支持肿瘤细胞的发展，如程序性细胞死亡蛋白1（PD-1）、程序性死亡配体1（PD-L1）和细胞毒性T淋巴细胞相关蛋白4（CTLA-4）。PD1和PD-L1可以相互作用，使肿瘤细胞继续生长。为了克服这一事件，PD1和PD-L1抗体可以阻止它们的相互作用，并允许细胞毒性T细胞杀死肿瘤细胞。此外，靶向抗原的密度、肿瘤微环境、供者T细胞的状态和疾病的特征都会影响CAR-T细胞的识别或杀伤，这些都影响CAR-T细胞治疗的反应[28]。本研究T细胞免疫治疗组的高缓解率提示，更换靶点（从CD19转为CD20）的T细胞免疫疗法可能克服肿瘤细胞CD19丢失或调动新的T细胞克隆来克服既往CD19 CAR-T细胞克隆功能耗竭所致的耐药。CD20在B细胞淋巴瘤中广泛表达且相对稳定，在CD19 CAR-T治疗后仍可能保持表达，这为靶向CD20的T细胞免疫治疗提供了理论基础。Frank等[29]发现CD22 CAR-T在CD19 CAR-T失败患者中有效，提示更换靶点可绕过CD19丢失及CAR-T耗竭导致的耐药。C-CAR066研究同样证实了抗CD20 CAR-T在CD19 CAR-T失败LBCL中的高缓解率[18]。Xue等[30]的研究中也显示CD19 CAR-T治疗失败患者的挽救治疗中，CD20-SD-CART组的整体应答率（27.8％）也显著高于非CART组（7.9％，*P*＝0.03）。此外，本研究中2例接受抗CD3×CD20双特异性抗体（格菲妥单抗）的患者也获得深度缓解，一项开放多中心Ⅰb/Ⅱ期临床研究评估了格菲妥单抗联合维泊妥珠单抗治疗大B细胞淋巴瘤的治疗效果，其中28例既往接受CAR-T细胞治疗患者的ORR为75％，一半的患者获得CR[22]。这也提示双特异性抗体同样有效，且具有即用型、制备周期短等优势。再次输注CAR-T细胞是另一种挽救策略，但既往研究显示，第二次输注相同靶点的CAR-T细胞疗效有限：TRANSCEND-NHL-001研究中liso-cel再治疗ORR仅19％[2]，ZUMA-1研究中axi-cel再治疗ORR为54％但中位DOR仅81 d[4]。更换靶点（如CD22 CAR-T）在CD19 CAR-T失败后的ORR为68％，CR率为53％[29]，略低于本研究的抗CD20 T细胞免疫治疗，可能与靶点特性、患者人群差异有关。本研究结果进一步支持更换靶点的T细胞免疫疗法作为CAR-T失败后优选策略。

本研究表明靶向CD20 T细胞免疫治疗的安全性特征是可接受和可控的。最常见的不良事件是血液学毒性和CRS。虽然有10例（83.3％）患者发生了CRS，但均为轻度CRS，无ICANS发生，均未使用托珠单抗和类固醇激素治疗。CRS的发生率和等级低于患者既往使用的靶向CD19 CAR-T细胞疗法。基于本研究及文献，建议CAR-T失败后应尽早进行靶向CD20的T细胞免疫疗法，避免无效化疗导致体能下降。尤其对于进展迅速、肿瘤负荷高的患者，推荐直接使用抗CD20 CAR-T或双特异性抗体。抗CD20 CAR-T适用于年轻、器官功能良好、可等待细胞制备（2～3周）的患者；双特异性抗体适用于需要立即治疗、无法采集足够T细胞或有中枢神经系统累及的患者（本研究中2例双抗均有效，包括中枢复发）。

本研究有一些局限性。这是一项回顾性研究，两组之间可能存在选择偏倚，因此在将其结果与其他挽救性治疗进行比较时需要谨慎。治疗前的CD20表达仅通过免疫组织化学进行定性评估，因此无法确定CD20表达强度对疗效的影响。此外，本研究样本量小，无法分析影响疗效的因素。另外，未检测到先前CAR-T细胞残留，因此未分析先前CAR-T的存在对T细胞功能的影响。

综上所述，本研究初步结果显示，非靶向CD20 T细胞免疫疗法包括抗体药物偶联物、分子靶向药物联合多药化疗在抗CD19 CAR-T疗法失败后的r/r DLBCL的治疗中的疗效是有限的，而靶向CD20 T细胞免疫疗法在抗CD19 CAR-T疗法失败后的r/r DLBCL中疗效显著，具有良好的安全性特征和高且持久的缓解，为既往抗CD19 CAR-T疗法失败的患者提供了一个有希望的治疗选择。
